# Generation of a novel mouse model of nemaline myopathy due to recurrent *NEB* exon 55 deletion

**DOI:** 10.21203/rs.3.rs-5456324/v1

**Published:** 2024-12-17

**Authors:** Zachary Coulson, Justin Kolb, Nesrin Sabha, Esmat Karimi, Zaynab Hourani, Coen Ottenheijm, Henk Granzier, James J. Dowling

**Affiliations:** 1 Program for Genetics and Genome Biology, Hospital for Sick Children, Toronto, ON, CAN; 2 Department of Molecular Genetics, University of Toronto, Toronto, ON, CAN; 3 Department of Physiology, University of Arizona, Tucson, Arizona, USA; 4 Department of Physiology, Amsterdam UMC, Amsterdam, North-Holland, NL

**Keywords:** nebulin, nemaline myopathy, pseudoexon, transcript stabilization, CRISPR, phenotyping

## Abstract

Biallelic pathogenic variants in the nebulin (*NEB*) gene lead to the congenital muscle disease nemaline myopathy. In-frame deletion of exon 55 (ΔExon55) is the most common disease-causing variant in *NEB*. Previously, a mouse model of *Neb*^ΔExon55^ was developed; however, it presented an uncharacteristically severe phenotype with a near complete reduction in *Neb* transcript expression that is not observed in *NEB* exon 55 patients.

We identified by RNA sequencing that the cause of this unexpectedly severe presentation in mice is the generation of a pseudoexon containing two premature termination codons (and promoting nonsense mediated decay) at the *Neb* exon 55 deletion site. To prove that this is the cause of the loss of *Neb* transcript, and to generate a more faithful model of the human disease, we used CRISPR gene editing to remove the pseudoexon sequence and replace it with human intron 54 sequence containing a validated cas9 gRNA protospacer. The resulting “hmz” mice have a significant reduction in pseudoexon formation (93.6% reduction), and a re-introduction of stable *Neb* transcript expression. This new model has the characteristic features of nemaline myopathy at the physiological, histological, and molecular levels. Importantly, unlike the existing exon 55 deletion mice (which die by age 7 days), it survives beyond the first months and exhibits obvious signs of neuromuscular dysfunction. It thus provides a new, robust model for studying pathomechanisms and developing therapies for *NEB* related nemaline myopathy.

## Background

Nemaline myopathy (NM) is a rare congenital skeletal muscle disease that affects approximately 1:50000 people [1]. NM patients typically present in infancy with muscle weakness and reduced muscle tone; in severe cases, NM can lead to early death [2]. To date, mutations in 13 genes have been identified to cause NM, with the most common gene involved being nebulin (*NEB*) [2–4].

Recessive mutations in *NEB* account for > 50% of cases of nemaline myopathy [5]. In-frame deletion of the 105 base pair exon 55, either in homozygosity or in heterozygosity with a second pathogenic variant, is the most cause of *NEB* related NM [6]. It results most typically in congenital onset nemaline myopathy, and at the molecular level produces normal *NEB* transcript levels but reduced Nebulin protein expression [2,7]. *NEB* encodes Nebulin, a giant structural protein involved in the assembly of the sarcomere, the skeletal muscle’s contractile structure. Among other functions, it serves as a molecular ruler, establishing the length of the sarcomere thin filament [8]. Key histopathological features of *NEB-*related NM include the formation of nemaline rod protein aggregates in skeletal muscle tissue, shortening of sarcomere thin filament length (TFL), and reduction of muscle force generation [6,9,10]. The deletion of *NEB* exon 55 destabilizes nebulin’s binding to the thin filament and is predicted to lead to its degradation [11].

In 2013, we (Granzier) established an *Neb*^ΔExon55^ mouse model; however, this model suffers from an uncharacteristic reduction in nebulin expression at the transcript level that is not seen in humans [12]. The result is a severe motor phenotype and early perinatal mortality that is similar to what is observed in *Neb* knockout mice [13]. This is more severe than the human molecular and clinical phenotypes and presents a challenge for pathomechanistic studies and therapeutic testing. It thus limits the translatability of this model for human *NEB* related NM.

To overcome these challenges, we sought to identify the underlying cause of the reduction in *Neb* transcript in exon 55 deletion mice, and to correct it in order to ameliorate the severe phenotype. Through total bulk RNA sequencing (i.e. RNAseq), we identified transcript abnormalities, including inclusion of a pseudoexon with premature stop codon(s)), that account for the reduction of *Neb* transcript in this model. We then generated an amended, minimally humanized *Neb*^ΔExon55^ model termed Hmz*-Neb*^ΔExon55^. Our Hmz*-Neb*^ΔExon55^ model has restored transcript stability leading to wild type (WT) levels of mutant *Neb* RNA but reduced Neb protein expression, similar to what is observed in *NEB* exon 55 deletion patient muscle. It presents with characteristic NM features at the physiological, histological, and molecular levels. This novel Hmz*-Neb*^ΔExon55^ model thus represents an important advancement in *NEB-*related NM research, providing a robust model to study *Neb* related NM and to develop and test new therapies.

## Materials and methods:

### Model generation and genotyping:

**Figure 1:**
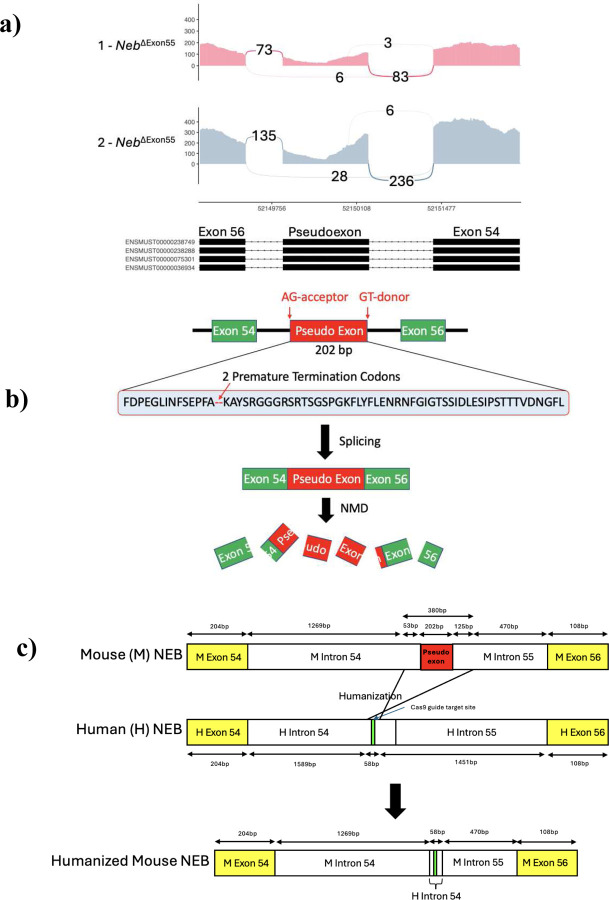
Pseudoexon identification and Hmz-*Neb*^ΔExon55^ model generation. (**A**) RNAseq Sashimi plots from *n=*2 *Neb*^ΔExon55^ mice. Junction reads spanning exons 54–56 indicate that there is ~94% pseudoexon inclusion in *Neb*^ΔExon55^ mice. (**B**) Cartoon depiction of *Neb*^ΔExon55^ pseudoexon depicting the location of cryptic splice sites leading to the pseudoexon being spliced into the mature mRNA. Translated amino acid sequence of the pseudoexon depicting the presence of two premature termination codons (PTC). The presence of these PTCs can lead to transcript degradation through nonsense mediated decay (NMD) and would explain why there is a reduction in *Neb* transcript levels in the *Neb*^ΔExon55^ mice. (**C**) Schematic of Hmz-*Neb*^ΔExon55^ model development whereby 380bp of sequence encompassing the pseudoexon is replaced with a short 58bp human intron 54 sequence containing a validated cas9 gRNA protospacer sequence. The resulting allele has a slightly shorter intronic sequence than the original and no longer contains the pseudoexon sequence that was being spliced in.

The Hmz*-Neb*^ΔExon55^ mouse was generated in collaboration with The Centre for Phenogenomics (TCP) using CRISPR-Cas9 to delete 380 bp of intron sequence from the exon 55 deletion site of the previous *Neb*^ΔExon55^ mouse model (Figure 1c). Genotyping was performed using two PCR reactions to amplify either the WT or humanized deletion allele.

Primers:

WT allele

Forward: GCATTCTTGCTCTTTCTTGTATGG

Reverse: GAAAGGAACTCTGTCCTCTGG

Hmz*-Neb*^ΔExon55^ allele

Forward: AAGCTAGGGTGTTTGAGTCTCTTC

Reverse: GACTGGAGCAACACACATTGTAC

### RNA sequencing:

For the previous *Neb*^ΔExon55^ model, whole hind limb muscle was harvested at PN 2 and for Hmz*-Neb*^ΔExon55^ gastroc tissue was harvested at ~2 months of age and placed on dry ice before being stored at −80C for later RNA extraction. A Qiagen RNeasy Fibrous Tissue RNA extraction kit was used to isolate RNA from gastroc tissue. RNA samples were sent to The Centre for Applied Genomics (TCAG) where they were prepared and sequenced. RNA underwent poly(A) enrichment and samples were prepped with New England Biolabs Next Ultra II Directional RNA-Seq for sequencing. RNA preps were sequenced using the Illumina NovaSeq 6000 platform at a depth of 100 million reads per sample. *Neb* gene read data was mapped to the GRC-m39 mouse genome assembly and analyzed in Integrated Genome Viewer (IGV). Custom mouse genomes were created to include either the residual FRT + vector sequence from the previous *Neb*^ΔExon55^ model or the minimally humanized deletion site in the new Hmz*-Neb*^ΔExon55^ model to accurately map the pseudoexon sequence. Sashimi plots were generated in ggsashimi according to Breschi et al. (2018) [14]. Junctions reads with a frequency below 3 were discarded. Transcript per million (TPM) junction reads encompassing *Neb* exons 54 to 56 were used to compare the proportion of transcript with a pseudoexon to that without.

### General Phenotyping

#### Animal Care and Monitoring

All animal procedures were performed in compliance with the Animals for Research Act of Ontario and the Guidelines of the Canadian Council on Animal Care. All protocols and procedures were pre-approved by The Centre for Phenogenomics (TCP). Animals were housed in appropriately temperature and light cycle controlled specific pathogen free conditions, in cages containing food, unlimited access to water, bedding material, and a plastic handling tube.

Daily welfare assessments were performed according to TCP standard procedure to determine if humane endpoint was met. No unexpected outcomes were observed leading to humane endpoint.

#### Body Weight

Mouse body weights were measured twice weekly until ~2-months of age whereafter they were measured weekly until they reached endpoint.

#### Open field

Mice were allowed to acclimatize to the testing room in their home cages for 30 minutes prior to beginning open field testing. After 30 minutes, animals were placed in 43.5×43.5cm open field chambers centrally illuminated to 250 lux. Chambers contained 16 beam IR rays (X, Y, and Z axes) to monitor mouse movement. Mouse movement was monitored over 20 minutes using Med Associates activity monitoring software. Horizonal movement data was collected according to X and Y axis beam breaks and rearing was measured according to the number of Z axes beam breaks.

### Cryosectioning

TA and Quad muscle were harvested, and flash frozen in isopentane cooled by liquid nitrogen. Frozen muscle was stored at −80 ^o^C. 8µm horizontal and longitudinal sections were cut from the centre of frozen tissues at −30 ^o^C on a Leica CM 1860 cryostat and affixed to Fisherbrand Superfrost Plus microscope slides. Slides were stored at −80 °C for staining.

### Modified gomori trichrome

Staining was performed according to https://www.newcomersupply.com/product/trichrome-stain-solution-gomori-one-step-light-green. In brief, 8µm quadricep sections affixed to Fisherbrand Superfrost Plus slides were dried for 10 minutes, stained with 0.5% vector hematoxylin counterstain for 10 minutes then rinsed in tap water for 3 minutes. Slides were stained in gomori trichrome one step light-green (newcomer supply) at 39°C for 20 minutes. Slides were then rinsed with distilled water and differentiated in 0.25% acetic acid. Slides were dehydrated for 5 min in 95% ethanol followed by 5 min in 100% ethanol. Slides were cleared in xylene for 2 minutes then mounted with toluene before adding a glass cover slip and left dry overnight. Slides were imaged on Olympus BX43 light microscope at 40X magnification.

### Immunofluorescence

#### Dystrophin fiber size IF and nebulin and α-actinin double IF

Slides with muscle sections were brought to room temperature and dried for 2 min. tissues were fixed with cold 4% PFA at room temperature for 20 min. Slides were then washed 3 times in wash buffer (1x TBS, 0.1% Triton X-100, 0.1% Tween-20) in a staining jar on a tilter table for 5 min. Tissue was blocked in blocking buffer (1x wash buffer, 1% bovine serum albumin, 10% goat serum) for 1 hour at room temperature in a moisture chamber. Slides were then incubated with 1/100 dilutions of primary antibody (Myomedix Neb N-term #6969, Abcam α-actinin A7811, Abcam dystrophin Ab15277) overnight at 4°C. The following day, slides were washed again following the previous wash step and then incubated with 1/1000 dilution of secondary antibody (Alexa fluor 488 (green) or 555 (red)) in blocking buffer at room temperature for 1 h in the dark. Slides were washed again following previous wash steps in the dark. ProLong Gold Antifade with DAPI mountant was added to the tissue and then sealed with a coverslip and left to dry in the dark for 24 h at room temperature before imaging.

N=5–7 20X images per sample were taken from dystrophin stained slides on an Olympus BX43 microscope. Images were processed and fiber area and Ferets diameter was determined in ImageJ. Fibers with an area less than 300 were discarded to remove fiber assignment artifacts and fibers were binned by minimum Ferets diameter in GraphPad. 200X images from neb N-term and α-actinin co-stained slides were imaged on a Nikon A1R confocal microscope at a depth yielding maximal nebulin staining intensity.

#### Fiber typing IF

Staining was performed according to Luca J. Delfinis et al, (2022) [15]. In brief, slides with muscle sections were brought to room temperature and dried for 2 min. After drying, slides were treated with blocking buffer (wash buffer, 5% goat serum) for 1h at room temperature. Slides were then incubated with 1/25 dilutions of primary antibody (DSHB MHCI BA-F8, MHCIIa SC-71, MHCIIb BF-F3) in blocking buffer overnight at room temperature. The following day, slides were washed in wash buffer for 20 min at room temperature on a tilter table. Next the slides were incubated with 1/1000 dilution of secondary antibody (alexa fluor 350 IgG 2b (blue), 488 IgGI (green), 568 IgM (red) in blocking buffer for 1 h in the dark. After secondary staining slides were washed in wash buffer for 20 min at room temperature in the dark. ProLong Gold Antifade mountant was added to the tissue and then sealed with a coverslip and left to dry in the dark for 24 h at room temperature before imaging.

N=5–7 20X images were taken per sample from multi-MHC stained slides on an Olympus BX43 microscope. Fiber content was calculated manually in imageJ and pie charts and bar charts were generated in GraphPad.

### Transmission electron microscopy

Thin longitudinal TA slices were taken from freshly harvested TA muscle and submersed in fixative containing 2.5% glutaraldehyde and 0.1 M sodium cacodylate buffer. Tissue was kept at room temperature for 20 min and then placed at 4°C overnight. The following morning samples were brought to the Advanced Bioimaging Center (The Hospital for Sick Childen). Here 90 nm thick sections were prepared on an RMC MT6000 ultramicrotome and then stained with uranyl acetate and lead citrate. Sections were then imaged on a FEI Tecnai 20 TEM microscope at 6000X and 30000X magnifications.

### Protein analysis

#### Whole protein analysis gel

Following Methods was modified from Kiss B et al, (2020) [8]. Flash-frozen tissues were pulverized in liquid nitrogen and then solubilized in urea buffer [8 M urea, 2 M thiourea, 50 mM tris-HCl, 75 mM dithiothreitol with 3% SDS, and 0.03% bromophenol blue (pH 6.8)] and 50% glycerol with protease inhibitors (0.04 mM E64, 0.16 mM leupeptin, and 0.2 mM phenylmethylsulfonyl fluoride) at 60°C for 10 min (Hidalgo, et. al., 2009) [16]. Solubilized samples were centrifuged at 13,000 RPM for 5 min, aliquoted, flash-frozen in liquid nitrogen, and stored at −80°C. Nebulin expression analysis was performed on solubilized samples using a vertical SDS-agarose gel system (Hoefer SE600). 1% gels were run at 15 mA per gel for 3hrs, then stained using Coomassie brilliant blue, and scanned using a commercial scanner. The scanned gels were subsequently analyzed with One-D scan (Scanalytics) and the optical density (OD) of Titin, Nebulin, and myosin heavy chain (MHC) was determined as a function of loading volume (in a range of six volumes). The slope of the linear relationship between OD and loading was obtained for each protein to quantify expression ratios. Nebulin and Titin expression levels were normalized to the MHC content, with final results normalized to the mean value of the MHC WT samples (Gineste, et. al., 2020) [17].

#### Nebulin western blotting

Solubilized samples were run on 0.8% SDS-Agarose gels and transferred onto polyvinylidene difluoride membranes using a semi-dry transfer unit (Trans-Blot Cell, Bio-Rad). Blots were stained with Ponceau S to visualize the total protein transferred. Blocking, detection with infrared fluorophore-conjugated secondary antibodies, and scanning followed recommendations for Odyssey Infrared Imaging System (LI-COR Biosciences). The following primary antibodies were used for Western Blotting: anti-nebulin N-terminal (1:1000; rabbit polyclonal; no. 6969, Myomedix). Protein expression was normalized to the MHC Ponseau S signal.

### Intact muscle mechanics

Methods adapted from Li F et al., (2015) and Brynnel A et al., (2018) [18,19]. Intact muscle mechanics was performed using the Aurora 1200A *ex vivo* test system that has been described previously (Labeit, et. al., 2010 and Ottenheijm, et. al., 2009) [20,21]. Briefly, muscles were attached between a combination servomotor-force transducer and fixed hook via silk suture in a bath containing oxygenated (95%/5% O_2_/CO_2_) Ringer solution (137 mM NaCl, 5.0 mM KCl, 1.0 mM NaH2PO4*H20, 1.0 mM MgSO4 * 7H20, 2.0 mM CaCl_2_ * 2H20, 24.0 mM NaHCO3, 11.0 mM glucose, pH 7.4, 30°C. Optimal current was determined using twitches (pulse duration of 200 μs with biphasic polarity), under light tension and set 50% beyond what is required to induce a maximum twitch force. The optimal length (*L*_0_) was determined by adjusting muscle length until a maximal twitch force was produced. Active force was determined from a force–frequency protocol. The Sol muscle was stimulated at incremental stimulation frequencies 1, 5, 10, 20, 40, 60, 80, 100 and 150 Hz waiting 30, 60, 60, 90, 120, 120, 120, 120 and 120 s, respectively, in between each stimulation. The EDL protocol matched that of the Soleus, except for an additional force measurement at frequencies of 200 and 250Hz. Muscle fatiguability was also measured by stimulating the soleus with a 40Hz tetanus every 3 seconds for 74 repetitions. EDL fatigue was measured the same way using 60Hz tetani. Measured force in mN were normalized by the physiological cross-sectional area (PCSA) of the muscle. The PCSA of the EDL and Soleus muscles were determined by using the measured muscle mass, muscle length, and taking the pennation angle of the fibers and the fiber length to muscle length ratio into account (Lieber and Ward, 2011) [22]. The PCSA was calculated as: $\text{PCSA}\left({\text{cm}}^{\text{2}}\right)=\frac{\text{muscle}\;\text{mass}\;\left(\text{g}\right)*\text{cos}\left(\theta \right)}{\rho \left(g\;cm^{-3}\right)*fiber\;lenth\;\left(cm\right)}$ (θ is the pennation angle and ρ is the physiological density of muscle).

From the force-frequency data, the maximal force produced, the minimal force produced, the time it takes to reach maximal force, the time the muscle takes to relax, and the frequency required to reach ½ of the maximal force can be extrapolated by fitting the force-frequency curve. The force-frequency curve was fit using the sigmoidal equation:

$$P_{0}\left(F\right)=P_{0min}+\left(P_{0\max}-P_{0\min}/\left\{1+exp\left[\frac{F_{half}-F}{k}\right]\right\}\right)$$

obtained from Prosser et al., 2011 where P0min gives the minimum specific force, P0max gives the maximum specific force, ${\text{F}}_{\text{half}}$ defines the frequency where ${\text{P}}_{\text{0}}\text{=}0.5$ of $P_{0max}$ and 1/k is a measure of the steepness of the $P_{0}$ vs. F relationship [23]. The curves for the different genotypes were also tested for significance using an extra sum of squares F-test. For fatigue, an index was used, where the average of the last 5 values measured were divided by the average of the first 5 values.

### Thin filament length measurements

Muscles were rapidly excised and placed in relaxing solution (in mM: 20 BES, 10 EGTA, 6.56 MgCl_2_, 5.88 NaATP, 1 DTT, 46.35 K-propionate, 15 creatine phosphate, pH 7.0 at 20°C) with 1% (w/v) Tritron X-100 and protease inhibitors for overnight on a 2D rocker at 4°C. The solution was then replaced with fresh relaxing solution (without Triton) followed by 5 h in 50% glycerol/relaxing solution before storing at −20°C. Skinned muscles were placed in a sylgard dish containing 50% glycerol solution and dissected into fiber bundles. The ends of the bundles were attached to aluminum T-clips and the solution replaced with fresh relaxing solution. Bundles were stretched ∼30% of their base length. Relaxing solution was then replaced with 4% formaldehyde solution and muscles were fixed for overnight. After fixation, muscles were washed with phosphate buffer saline (PBS) and embedded in Tissue-Tek O.C.T.compound (Ted Pella Inc) and stored at −80 °C. The O.C.T. embedded specimen was sectioned into 5 µm thick (Microm HM 550; Thermo Scientific) and placed on Super Frost Plus microscope slides. Fixed tissues were permeabilized again with 0.2% Triton X-100 in PBS for 20 min at room temperature on a light box to bleach out the background fluorescence. Washed with 1X PBS then incubated overnight at 4C in dark humidity chamber with Alexa Fluor 488-conjugated Phalloidin (for actin staining 1:1000, A12379, Life Technologies) in PBS. The tissues were washed with PBS for 15 min at room temperature, followed by 2 rapid washes with ddH20. Coverslips were mounted onto slides with Aqua Poly/Mount (Polysciences Inc.). Images were captured using a Deltavision RT system (Applied Precision) with an inverted microscope (IX70; Olympus), a ×100 objective, and a charge-coupled device camera (CoolSNAP HQ; Photometrics) using SoftWoRx 3.5.1 software (Applied Precision). The images were then deconvolved using SoftWoRx. An average of 10 areas was observed for each tissue section. Thin filament lengths and sarcomere lengths were obtained from deconvolved images of EDL muscles stained with a fluorescently conjugated phalloidin antibody. Deconvolved images were reopened in ImageJ (http://rsb.info.nih.gov/ij), then the 1D plot profile was calculated. The plot profile was analyzed using Fityk 0.13.1(http://fityk.nieto.pl). A custom ‘rectangle + 2 half Gaussian’ function was used for analyzing phalloidin-stained images that consisted of a rectangle that was flanked by two half Gaussian curves. To account for actin overlapping in the Z-disk which creates a small bump in the center of the rectangle, the center points within the rectangle fit were de-activated. This improved the subsequent fit for the ‘rectangle + 2 half Gaussian’ function. Thin filament length was calculated as half the width of the rectangle plus half the width of the Gaussian fit at half maximum height. SL was calculated from the distance between the centers of two adjacent Gaussian fits. We analyzed a large number of images and determined thin filament length within the SL range of 2.4–2.8 µm. WT and HOM EDL fiber bundles from N=3 male mice, 2 fiber bundles per animal.

### Statistical Analysis

Unless otherwise specified, the statistical analysis used includes either a two-tailed Student t-test (two-group single variable comparison) or one-way ANOVA (multiple-group single variable comparison) where relevant to determine the differences in group means.

## Results:

### Identification of a pseudoexon as the cause of transcript reduction in exon 55 deletion mice

The previously generated *Neb*^ΔExon55^ model has an unexpected reduction in nebulin transcript levels, with a corresponding complete reduction in protein expression [12]. To determine the cause, we performed bulk RNA sequencing on hind limb muscle RNA extracts from (*n=*2) 2-day old homozygous (HOM) and wild type (WT) *Neb*^ΔExon55^ mice. By analyzing the resulting Neb transcript(s), we identified a novel 202 bp pseudoexon transcribed from the exon 55 deletion site in the *Neb*^ΔExon55^ mice (Figure 1a). Of transcript reads that mapped to this locus of *Neb*, ~94.5% contained the pseudoexon (Figure 1a). Pseudoexons represent the incorporation of intronic material into the mature mRNA, and most commonly lead to introduction of premature stop codon(s) and resulting degradation of the transcript due to nonsense mediated decay [24–26]. In the sequence of this *Neb* pseudoexon, we identified two premature termination codons (PTCs) (Figure 1b). There is a corresponding significant, large magnitude reduction in *Neb* RNA levels, consistent with nonsense mediated decay of the pseudoexon containing transcripts.

### Creation of a novel humanized *Neb* exon 55 deletion mouse model

We hypothesized that changing the intronic sequence that produces the novel pseudoexon would result in production of a stable transcript and thus more closely model the impact of the human *NEB* exon 55 deletion. To accomplish this, we designed a new model using CRISPR-Cas9 mediated homology directed repair to excise 380 bp of intronic sequence encompassing the pseudoexon and replace it with a 58 bp human intron 54 sequence fragment. The inserted human intron 54 sequence also contains a human cas9 sgRNA protospacer sequence site for future exon 55 gene editing based repair experiments (Figure 1c). We predicted that deleting this portion of the gene would abrogate pseudoexon formation and named this model the minimally humanized *Neb*^ΔExon55^ mouse (Hmz*-Neb*^ΔExon55^) (Figure 1c).

**Figure 2:**
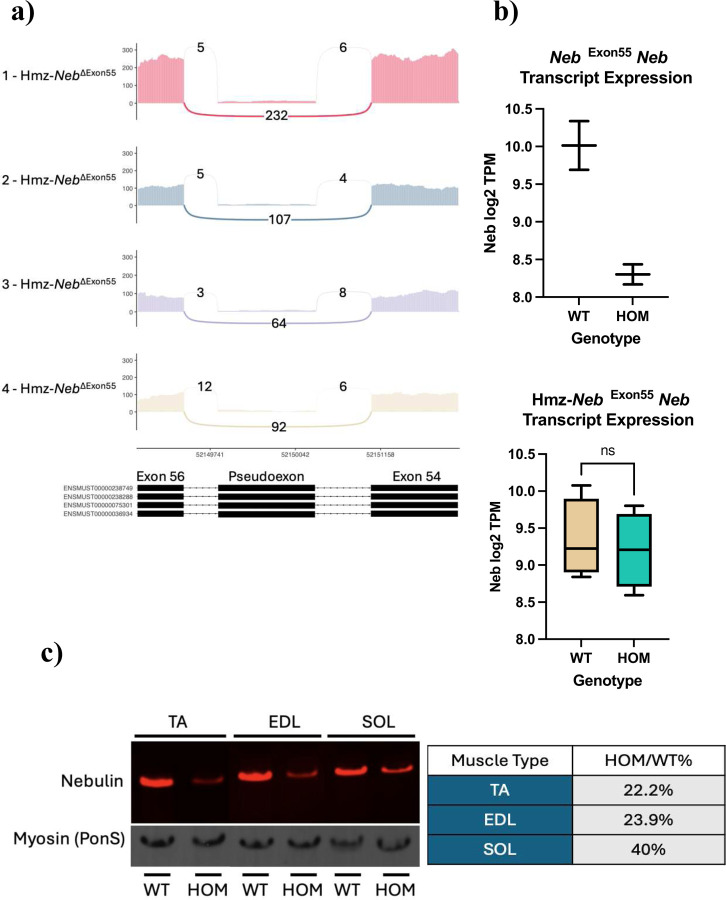
*Neb* transcript restoration and protein expression. (**A**) RNAseq Sashimi plots from *n=*4 Hmz-*Neb*^ΔExon55^ HOM animals encompassing *Neb* exons 53–57. Junction reads spanning exons 54–56 indicate that there is a reduction to ~6.4% in pseudoexon inclusion in Hmz-*Neb*^ΔExon55^ mice, with 94% of transcripts favoring complete exon skipping of exon 55 with no additional intronic sequence. (**B**) RNAseq comparing *Neb* transcripts per million (TPM) reads between *n*=2 WT and HOM *Neb*^ΔExon55^ mice and *n*=4 WT and HOM Hmz-*Neb*^ΔExon55^ mice. Transcript reads indicate a decrease in HOM *Neb*^ΔExon55^ mice *Neb* transcript levels and no significant change in Hmz-*Neb*^ΔExon55^ mice *Neb* transcript levels. (**C**) Western blot with an antibody to the N-terminus of nebulin comparing Hmz-*Neb*^ΔExon55^ WT and Hmz-*Neb*^ΔExon55^ tibialis anterior (TA), extensor longus digitorum (EDL), and soleus (SOL) nebulin protein expression normalized to PonceauS (Pon(S)) myosin heavy chain (MHC) staining. *n*=3 Hmz-*Neb*^ΔExon55^ mice see a reduction to 22.2% (TA), 23.9% (EDL), and 40% (SOL) WT nebulin protein expression levels.

RNAseq on RNA extracts from 2.5 month old gastrocnemius (gastroc) tissue (*n=*4) indicates that our Hmz*-Neb*^ΔExon55^ model has a significant reduction in pseudoexon containing *Neb* transcripts, from ~94.5% to ~6.4%, and no longer has a significant reduction in transcript expression levels (*P*=0.74) as compared to wild type littermates (Figure 2a,b). Western blot analysis with an antibody against nebulin’s N-terminus (with levels normalized to PonceauS MHC staining) indicates that the male Hmz*-Neb*^ΔExon55^ mice have a significant reduction in Neb protein expression as compared to wild type littermates across multiple skeletal muscle tissue types down to 22.2% in tibialis anterior (TA), 23.9% in extensor digitorum longus (EDL), and 40% in soleus (SOL) (Figure 2c). Follow up protein analysis by Coomassie stain on Hmz*-Neb*^ΔExon55^ TA indicates that there is a similar reduction to ~30% WT expression levels (Figure S1). Coomassie stain results indicate loss of the whole nebulin protein and not just a loss of its N-terminus. These levels are significantly increased as compared to the previous *Neb*^ΔExon55^ mice, where *Neb* protein expression is ~2% of WT levels [12]. This indicates that removal of the pseudoexon-promoting genomic sequence results in a stabilized *Neb* transcript with no pseudoexon, and that this transcript (which has only exon 55 deleted) results in increased (though still significantly reduced) levels of mutant nebulin protein expression as compared to the previous mouse model.

### Hmz*-Neb*^*ΔExon55*^ mice have reduced survival and impaired motor performance

**Figure 3:**
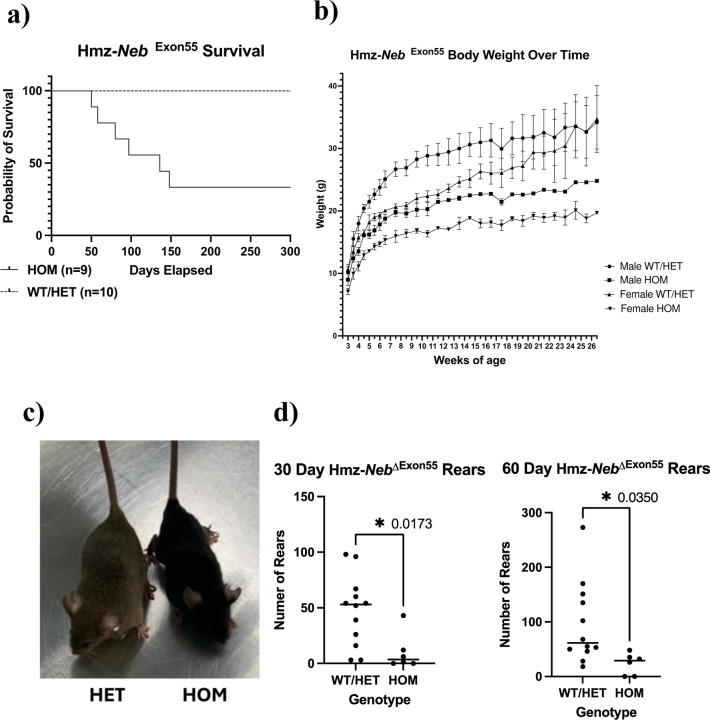
Hmz-*Neb*^ΔExon55^ survival and phenotyping. (**A**) Kaplan-Meier survival analysis comparing Hmz-*Neb*^ΔExon55^ *n*=10 pooled WT and HET mice to *n*=9 Hmz mice demonstrating that survival is reduced in the Hmz animals with a median survival of 139 days. (**B**) Body weight over time comparing Hmz-*Neb*^ΔExon55^ sex and age matched pooled WT and HET mice to Hmz mice demonstrating a reduction in male and female Hmz mice body weights that is first noted at 3 weeks of age and that persists until at least 26.5 weeks of age (age of last measurement). (**C**) Photomicrograph comparing a ~3-month-old HET (left-brown) and Hmz-*Neb*^ΔExon55^ mouse (HMZ, right-black) depicting HOM animals having a visibly distinct smaller stature. (**D**) Mouse rearing at 30 and 60 days of age measured through open field testing. Comparison is between Hmz-*Neb*^ΔExon55^ (*n=*6) mice and sex and age matched pooled WT and HET mice (30 day *n*=12, 60 day *n*=11). Hmz mice have a reduction of rearing at both 30 days of age and 60 days of age that is indicative of motor function deficits.

We performed a general phenotypic analysis of our new Hmz-*Neb*^ΔExon55^ model. Mice of each genotype were born at normal Mendelian ratios, but Hmz mice had reduced survival to a median age of 136 days (Figure 3a). Notably, Hmz*-Neb*^ΔExon55^ mice live significantly longer as compared to the previous non-edited HOM-*Neb*^ΔExon55.^ mice, which survive a median of 1 day and a mean of 4 days post birth. (Figure 3a, S2). Hmz*-Neb*^ΔExon55^ mice are phenotypically distinct from WT littermates by 4 weeks of age. Both male and female Hmz mice have reduced body weight compared to control littermates that persists over time (Figure 3b), with no observed decrease in tibia length (Figure S3). This indicates that this weight reduction that is likely the result of reduced muscle mass as opposed to whole body growth delay. Body mass reduction was visually obvious, as can be seen in images of 3-month-old Hmz*-Neb*^ΔExon55^ mice compared to WT littermates (Figure 3c).

As determined with open field analysis studies, Hmz*-Neb*^ΔExon55^ mice have significant motor impairments. Most notably, they have a significant reduction in rearing at 30 (*P*=0.0173) and 60 (*P*=0.0350) days of age when compared to control littermates (Figure 3d). Of note, reduced rearing is indicative of hind limb weakness in the Hmz*-Neb*^ΔExon55^ mice. Motor function is also qualitatively impaired, as Hmz*-Neb*^ΔExon55^ mice are less responsive to touch and have abnormal ambulation that includes slower movement and laboured gait (Figure S4).

### Hmz*-Neb*^*ΔExon55*^ mice present with altered muscle mechanics and contractile deficits

**Figure 4:**
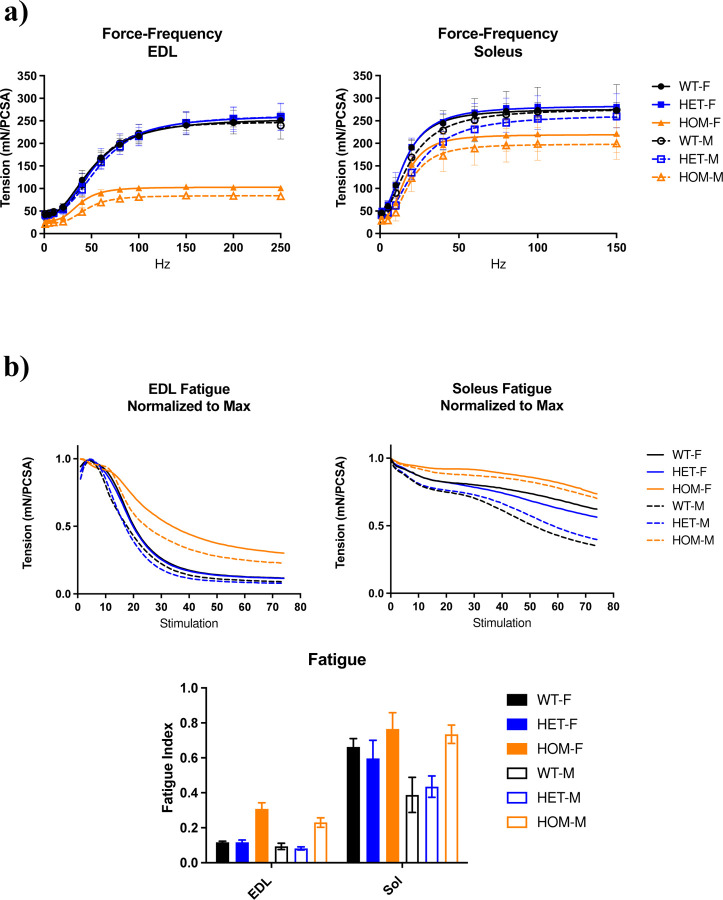
Hmz-*Neb*^ΔExon55^ intact muscle mechanics analysis. Intact muscle mechanics on male and female WT, HET, and HOM Hmz-*Neb*^ΔExon55^ EDL and SOL tissue. (**A**) Force frequency measurements on EDL and SOL muscle. Muscles were stretched to optimal length (*L*_0_) and then stimulated at incremental stimulation frequencies. Forces measure in mN and normalized to physiological cross-sectional area (PCSA) indicate that there is a large magnitude reduction in HOM EDL maximal force generation potential and a moderate reduction in HOM SOL maximal force generation potential that is more pronounced in male animals. (**B**) Muscle fatigue measurements on EDL and SOL muscle. Muscles were stretched to optimal length (*L*_0_) and then stimulated at constant 150Hz (SOL) and 200Hz (EDL) tetani every 3 seconds for 74 repititions. Fatigue measurements indicate that there is an increase in fatigue resistance that is higher in EDL than SOL muscle.

To confirm that the physiological movement deficits observed are related to skeletal muscle functional impairments, we performed *ex vivo* muscle mechanics studies on ~90-day old Hmz*-Neb*^ΔExon55^ EDL and SOL muscle. Hmz*-Neb*^ΔExon55^ mice have a dramatic reduction in EDL specific force generation potential and a moderate but non-significant reduction in SOL specific force generation compared to control littermates (Figure 4a). The EDL and SOL also see an increase in fatigue resistance compared to control littermates (Figure 4b). Both the impairments in force generation potential and increase in fatigue resistance appears more profound in males, which led us to focus our subsequent analyses in male animals.

### Hmz*-Neb*^*ΔExon55*^ mice have altered myofiber content, reduced muscle weight, and myofiber fiber size

**Figure 5:**
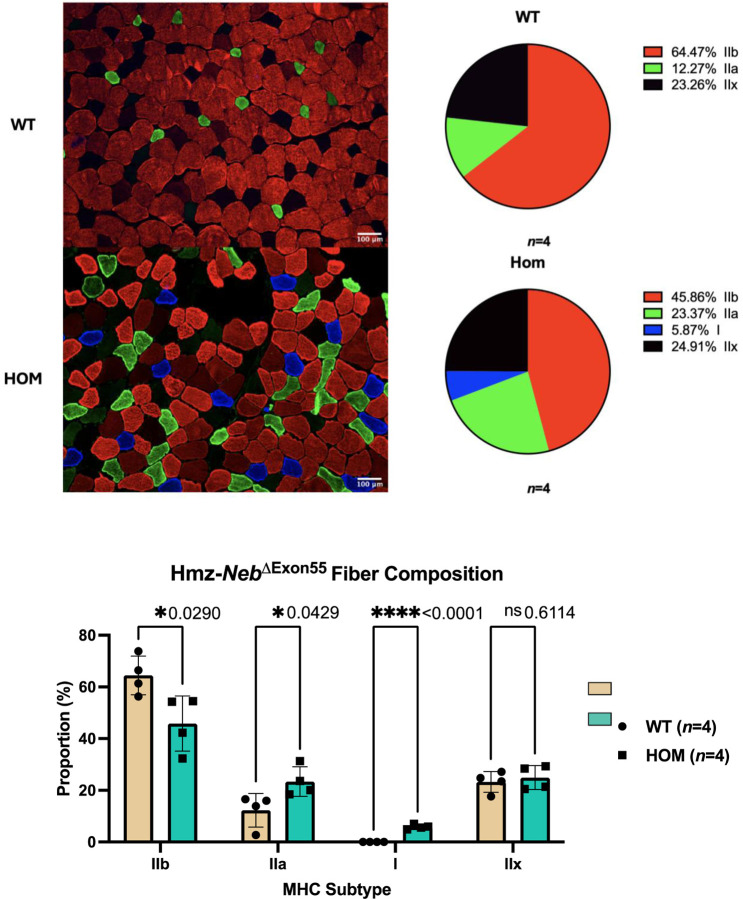
Hmz-*Neb*^ΔExon55^ TA fiber type composition shift. Comparing skeletal muscle fiber composition in (*n*=4) ~2-month-old Hmz-*Neb*^ΔExon55^ male mice TA muscle. Isopentane flash frozen horizontal TA sections were stained for myosin heavy chain (MHC) type IIb, IIa, and I protein. *n*=8 representative 20X magnification immunofluorescence images were taken per sample and quantified by hand in ImageJ for fiber content. HOM animals see a significant decrease (*P*=0.029) in type IIb fast fiber composition and a significant increase in type IIa (*P*=0.0429) and I (*P*<0.001) slow fiber composition with no significant (*P*=0.6114) change in type IIx composition.

**Figure 6:**
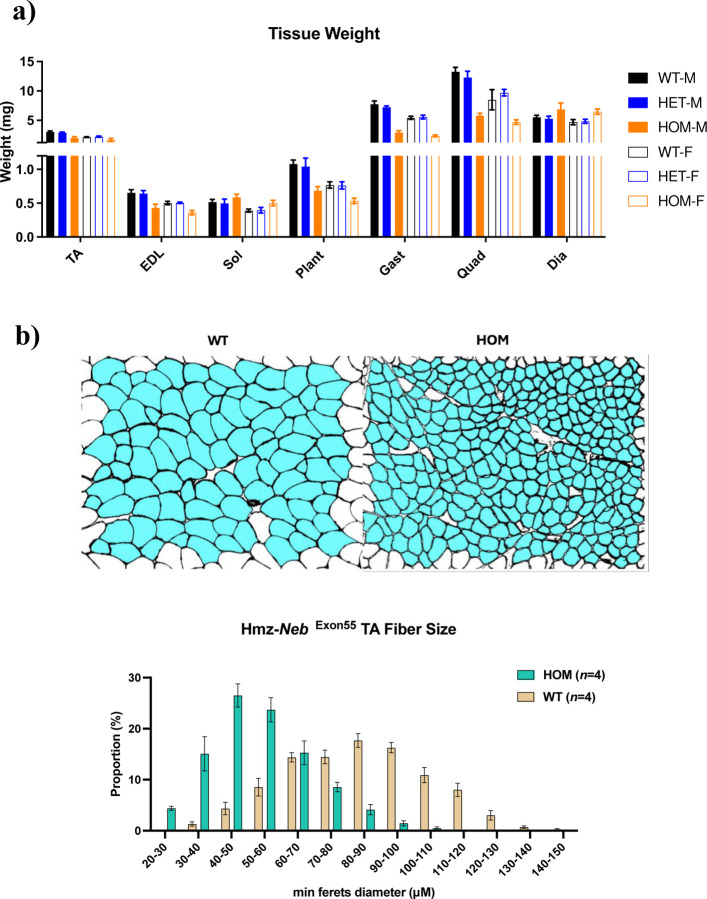
Hmz-*Neb*^ΔExon55^ muscle tissue weight and fiber size reduction. (**A**) Muscle tissue weights for ~3-month-old Hmz-*Neb*^ΔExon55^ WT, Het, and HOM mice. HOM animals have a reduction in tibialis anterior (TA), extensor digitorum longus (EDL), plantaris (Plant), gastrocnemius (Gast), and quadriceps (Quad) muscle compared to WT and HET littermates indicative of hypotrophy. Conversely, HOM animals have either no change or else a slight increase in soleus (SOL) and diaphragm (Dia) muscle weights compared to WT and HET littermates. (**B**) Measuring skeletal muscle fiber size in (*n*=4) ~2-month-old Hmz-*Neb*^ΔExon55^ male mice TA muscle. Isopentane flash frozen horizontal TA sections were stained for dystrophin, a membrane associated protein. *n*=8 representative 20X magnification immunofluorescence images were taken per sample and quantified in ImageJ for minimum Feret’s diameter. Imaging indicates a significant shift to smaller fiber size composition within HOM animals compared to WT.

The observed increase in fatigue resistance could in part be explained by a corresponding fiber type shift to type IIa and I MHC expressing fibers that are more fatigue resistant. To investigate this, we performed IF with antibodies against multi-MHC subtypes on cross sections of TA muscle. Comparison of Hmz*-Neb*^ΔExon55^ and WT littermates (*n*=4) indicated a reduction in type IIb fast glycolytic fibers (~64.47% to ~45.86%, *P=*0.029) and an increase in type IIa fast oxidative (~12.27% to ~23.37%, *P=*0.0429) and type I slow oxidative (0% to 5.87%, *P*<0.0001) fibers. Type IIx fiber composition was unchanged (~23.26% to ~24.91%, *P=*0.6114) (Figure 5). In addition to fiber type proportion changes, Hmz*-Neb*^ΔExon55^ mice have a reduction in tissue weight across several skeletal muscle groups including TA, EDL, plantaris, gastrocnemius, and quadriceps (Figure 6a). Reduced muscle weight was correlated with a quantitative reduction in muscle fiber size in Hmz*-Neb*^ΔExon55^ TA muscle (Figure 6b).

### Exon 55 deletion leads to the production of nemaline rods

**Figure 7:**
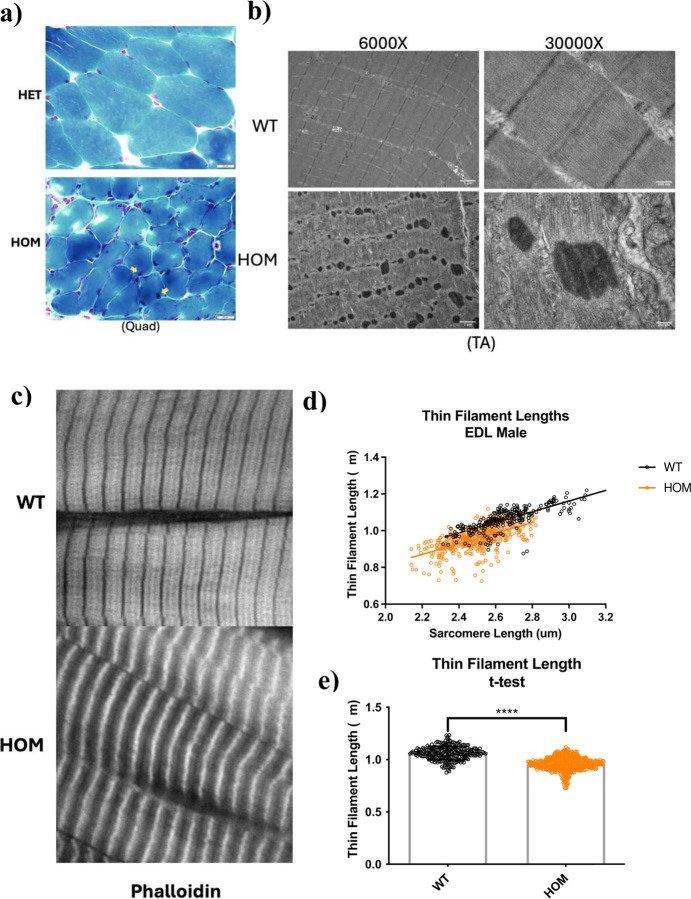
Hmz-*Neb*^ΔExon55^ nemaline rod formation and reduced thin filament length. (**A**) Modified gomori-trichrome stain on isopentane flash frozen Quad crossections from *n*=2 ~2-month-old Het and HOM male Hmz-*Neb*^ΔExon55^ mice indicates that HOM mouse fiber size is reduced with apparent nemaline rod formation (yellow arrow). (**B**) TEM imaging of glutaraldehyde fixed longitudinal TA sections from *n*=4 ~2-month-old WT and HOM Hmz-*Neb*^ΔExon55^ mice indicates clear nemaline rod formation throughout HOM tissue that is not present in WT. (**C**) Phalloidin staining ~3-month-old WT and HOM Hmz-*Neb*^ΔExon55^ thin filaments. (**D**) Thin filament length (TFL) measurements indicates that there is a shift to shorter TFL and sarcomeres in HOM animals. (**E**) WT/HOM t-test indicates that the TFL reduction from 1.06μm in WT to 0.96μm in HOM animals is significant (*P*<0.001).

The primary histopathological hallmark of *NEB* related NM is the formation of nemaline rods in skeletal muscle [6,9,10,27–29]. Nemaline rods are aberrant structures that emanate from the Z-disk of the sarcomere and are composed of a variety of sarcomere associated proteins such as α-actinin, actin, cofilin-2, myotilin, nebulin, telethonin, tropomyosin, and γ-filamin [3,30]. Modified Gomori trichrome staining on horizontal quadriceps sections (*n*=3) indicated the presence of muscle fibers containing nemaline rods in Hmz*-Neb*^ΔExon55^ mice (Figure 7a). As nemaline rods are most definitively shown with high magnification ultrastructural analysis, transmission electron microscopy (TEM) was performed on longitudinal TA sections. TEM indicated that the presence of numerous aggregates consistent with nemaline rods throughout Hmz*-Neb*^ΔExon55^ muscle (*n*=4) (Figure 7b). Rod size appeared variable with many rods exhibiting a filamentous structure and localizing to the sarcomere Z-disk (Figure 7b, S5). While rod formation is pervasive, sarcomere alignment appeared largely conserved except at locations with larger rods (Figure 7b).

### Thin filament length is reduced in in Hmz*-Neb*^*ΔExon55*^ mice

In addition to rod formation, thin filament length (TFL) reduction is another key structural marker of *NEB* related NM [10]. To measure TFL, we performed deconvolution IF imaging on Hmz*-Neb*^ΔExon55^ EDL muscle sections stained for phalloidin (Figure 7c). We focused on EDL because it had the most significant force production deficits from our mechanics studies (Figure 4). EDL TFL measurements indicate a significant reduction in TFL (*P*<0.0001) from 1.06μm in WT to 0.96μm in HOM Hmz*-Neb*^ΔExon55^ tissue (Figure 7d, e). Intriguingly, actin staining by phalloidin appears weaker, with a much broader and non-uniform intense band at the Z-disk consistent with the Z-disk thickening and Z-disk localized nemaline rod formation noted by TEM (Figure 7c).

### Nebulin IF demonstrates reduced and variable expression but with areas of normal localization

**Figure 8:**
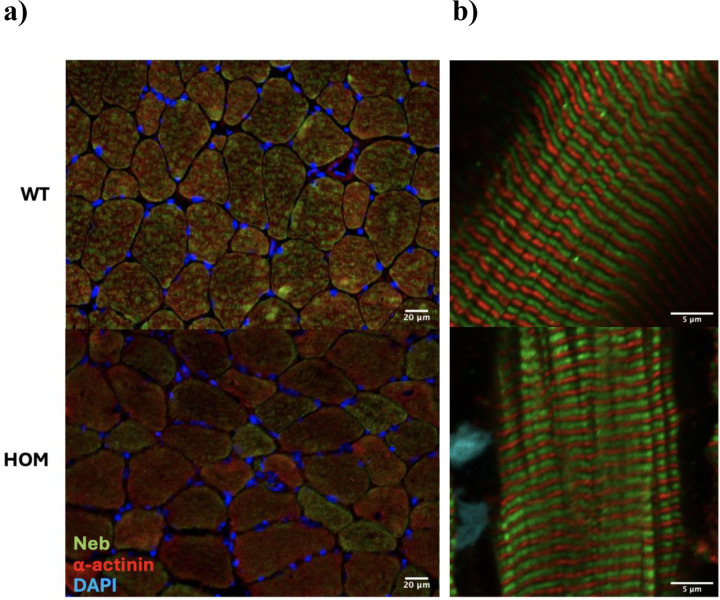
Nebulin distribution and localization. Double Immunofluorescence labelling for *Neb* N-terminus and α-actinin in *n*=4 WT and HOM male Hmz-*Neb*^ΔExon55^ TA muscle. (**A**) Cross section indicates the proportion of higher nebulin expressing fibers is reduced in HOM mice. (**B**) Confocal imaging of longitudinal sections demonstrates that nebulin’s localization is normal in HOM mice.

To examine nebulin’s distribution in muscle tissue and determine if its localization is impaired, we performed nebulin immunostaining. Double IF for nebulin’s N-terminus and α-actinin in *n*=4 Hmz*-Neb*^ΔExon55^ horizontal TA sections indicates that there is substantial inter-myofiber variability of nebulin expression (Figure 8a). Some fibers express nebulin similarly to WT, while the majority appear to have a visible reduction (Figure 8a), though with the detectable signal still properly localized to the thin filament (Figure 8b). This suggests that while the in-frame deletion of exon 55 destabilizes the nebulin protein, the protein that is produced still integrates into the sarcomere normally.

## Discussion

Since its initial description, the *Neb* exon 55 deletion mouse model has presented a mystery. Why are *Neb* transcript levels significantly reduced by a small in-frame deletion, particularly when levels are not altered in patients with the same mutation? In this study, we solve this mystery: exon 55 deletion in mice (as opposed to patients) results in the formation of a pseudoexon at the deletion site, and this pseudoexon introduces premature stop codons that ultimately leads to absence of *Neb* RNA and protein and a severe *Neb* related phenotype that resembles that of *Neb* knockout mice. We modified the deletion locus to abrogate pseudoexon formation and stabilize the *Neb* transcript, which has led to the generation of a new mouse model that now closely phenocopies what is observed in patients with *NEB* related nemaline myopathy due to homozygous exon 55 deletion. This includes normal transcript but decreased Neb protein levels, small whole-body size and impaired motor performance, reduced myofiber size, the presence of nemaline rods on muscle biopsy, and reduced thin filament length with resulting diminution in force generation.

### Significant reduction in Hmz-*Neb*^ΔExon55^ pseudoexon formation stabilises transcript

Pseudoexons have previously been reported as a disease driving mechanism and have been identified as a *NEB* related NM pathogenic driver [31,32]. In the case of *Neb*^ΔExon55^ mice, a pseudoexon is formed in the intronic region of the exon 55 deletion site that contains two PTCs. PTCs located in the central region of multi-exonic genes typically initiate nonsense mediated decay, which explains the significant reduction in *Neb* transcript observed in *Neb*^ΔExon55^ mice [25,26]. By deleting 380 bp of intronic sequence encompassing the pseudoexon site and replacing it with a 58 bp human intron 54 sequence, we were able to almost completely abrogate pseudoexon formation and restore *Neb* transcript stability.

### Transcript restoration results in a less severe and more characteristic reduction in nebulin protein

Restoring nebulin transcript levels leads to a corresponding increase in mutant nebulin protein expression, relative to *Neb*^ΔExon55^ mice. The protein level reduction we observed by N-term *Neb* western in the Hmz*-Neb*^ΔExon55^ TC (~22.2% of WT) and EDL (~23.9% of WT) is more in line with the reduction observed in *NEB*^ΔExon55^ patients (~12.5% of WT) when compared to the severe reduction to ~2% of WT levels observed in 5-day old *Neb*^ΔExon55^ mice [10,12]. Additionally, TA *Neb* protein quantification by gel analysis indicates a similar whole protein reduction in Hmz*-Neb*^ΔExon55^ mice (~30% of WT) to what is seen in patients (~20% of WT) [33]. The SOL on the other hand is more protected, with a reduction to ~40% of WT expression levels observed by N-term *Neb* western. A similar event was observed in an inducible *Neb* conditional knockout (cKO) mouse model, where the SOL sees a slower reduction in nebulin content over time compared to other tissues [18]. This is consistent with nebulin protein being more stable in the SOL, and is parsimonious with the data for our new Hmz*-Neb*^ΔExon55^ mice. Overall, the increase in nebulin protein levels in Hmz*-Neb*^ΔExon55^ mice compared to the previous *Neb*^ΔExon55^ is likely responsible for the more characteristic, human disease like phenotype we observe.

### Hmz-*Neb*^ΔExon55^ mice have extended survival compared to *Neb*^ΔExon55^ mice

In the previous *Neb*^ΔExon55^ model, there was a mean survival of 4 days of age, with fully penetrant mortality by 13 days of age. This parallels the mortality observed in a *Neb* KO model, where complete mortality was observed by 11 days of age [13]. This indicates that *Neb* is important for early life survival and suggests that the previous *Neb*^ΔExon55^ model’s phenotype is more in keeping with *Neb* KO. Our new Hmz-*Neb*^*ΔExon55*^ mice have a significant extension of life, attributable to the increased nebulin protein expression, a change that makes this model much more suited for pathomechanistic studies and future drug development work.

### Hmz-*Neb*^ΔExon55^ mice present with impaired motor function and muscle mechanics

Rearing observed through open field testing demonstrated hind limb function impairments in Hmz*-Neb*^ΔExon55^ mice as early as 30 days of age that persist until at least 60 days of age. Rearing has been used to demonstrate hind limb weakness in other skeletal muscle diseases, and motor function impairment is a primary symptom of *NEB*-related NM [2,34,35]. The presence of a rearing defect in Hmz mice is both consistent with persistent muscle weakness and representative of an outcome measure that is robust and quantifiable and thus suitable for future interventional studies.

Ex-vivo muscle mechanics analysis showed that Hmz*-Neb*^ΔExon55^ have a reduction in maximal specific force that is more pronounced in EDL muscle than SOL. Intriguingly, the degree of force reduction correlates with nebulin expression levels, as EDL muscle has a more dramatic loss of nebulin and force than the SOL. Skeletal muscle force generation reduction has been reported in several *NEB* ΔExon55 patients along with reduced calcium sensitivity and impaired cross-bridge cycling dynamics [10,28,33]. While EDL and SOL muscle had impaired force production, fatigue resistance was increased. This indicates that Hmz*-Neb*^ΔExon55^ mouse muscle is able to produce enduring contraction while under constant stimulation. An increase in muscle fatigue resistance was previously observed in the cKO *Neb* model and is consistent with fiber type composition shift to slower type I and IIa contracting fibers we observed in Hmz*-Neb*^ΔExon55^ TA muscle [17,18]. While the driver(s) of this fiber type composition shift are incompletely understood, the change is consistent with a similar shift to slow fibers observed in *NEB* related NM patients [29,33].

### Hmz-*Neb*^ΔExon55^ mice present with reduced body weight and impaired skeletal muscle development

Skeletal muscle hypotrophy and atrophy are commonly observed in *NEB* related NM patients [3,27,36]. In our model, we observe an overall reduction in body weight attributable to diffuse skeletal muscle hypotrophy, and a clear reduction in fiber size without redundant basal lamina that is also indicative of hypotrophy [3]. Of note, the SOL and diaphragm have increased weight, a finding which is consistent with hypertrophy. Hypertrophy in these muscles has previously been observed in the *Neb* cKO mouse model and a compound heterozygous *Neb* mouse model, where it was hypothesized to occur as a compensatory mechanism to the hypotrophy of synergistic muscle groups [17,18,37]. This is seen in Hmz*-Neb*^ΔExon55^ between the synergistic gastrocnemius and SOL muscle where the gasrtocnemius appears hypotrophic and the SOL appear potentially hypertrophic. Additionally, skeletal muscles with higher baseline contractile activity such as SOL and diaphragm have been reported to be more resistant to hypotrophy in *Neb* related NM [18].

### Hmz-*Neb*^ΔExon55^ mice present with hallmark nemaline rods and reduced thin filament length

The two key hallmark histopathological features of *NEB* related NM that have been observed in *NEB*^ΔExon55^ patients are the formation of nemaline rods and reduction in TFL [6,9,10,27–29]. Our Hmz*-Neb*^ΔExon55^ model has clear nemaline rod formation, with many of the rods having a filamentous structure as has been previously described [30]. Along with nemaline rod formation, we observed clear TFL reduction in Hmz*-Neb*^ΔExon55^ EDL muscle. Nebulin’s function as a molecular ruler for the thin filament has previously been described and nebulin has been shown to specify minimum TFL [8,38]. TFL reduction is likely a result of impaired TFL maintenance due to reduced nebulin function and availability in Hmz*-Neb*^ΔExon55^ mice. TFL reduction then leads to reduced actin-myosin crossbridge interactions resulting in impaired force generation [28,39].

## Conclusion

Our Hmz*-Neb*^ΔExon55^ model represents an important advancement in *NEB* related NM research as, to date, there was lack of a mouse model that accurately phenocopied the human disease. The severity of disease in the previous *Neb*^ΔExon55^ model limited the ability to study and characterize it and limited the translatability of findings to patients. We have demonstrated that our Hmz*-Neb*^ΔExon55^ mouse faithfully recapitulates *NEB*^ΔExon55^ related NM patient phenotypes, laying the groundwork for future studies into the pathomechanisms of *NEB*^ΔExon55^ related NM. In addition,, this new model will be ideal for evaluating therapeutic strategies. By stabilizing the *Neb* transcript and restoring protein expression we have significantly extended lifespan, broadening the window for therapy testing. We have also defined a spectrum of clinically relevant phenotypes that can be used as markers for measuring efficacy in response to future therapy treatment.

## Data Availability

All of the data that was collected and used to draw conclusions from this research is available and can be obtained by contacting the corresponding author upon reasonable request.

## List of abbreviations

Nebulin: NEB
nemaline myopathy: NM
thin filament length: TFL
wild type: WT
The Centre for Phenogenomics: TCP
The Centre for Applied Genomics: TCAG
Integrated genome viewer: IGV
transcripts per million: TPM
myosin heavy chain: MHC
optical density: OD
physiological cross-sectional area: PCSA
phosphate buffered saline: PBS
homozygous: HOM
heterozygous: HET
premature termination codon: PTC
nonsense mediated decay: NMD
tibialis anterior: TA
extensor digitorum longus: EDL
soleus: SOL
PonceauS: Pon(S)
plantaris: Plant
gastrocnemius: Gast
quadriceps: Quad
diaphragm: Dia
transmission electron microscopy: TEM
